# Some Observations on the Brain

**Published:** 1887-04

**Authors:** Henry C. Chapman

**Affiliations:** Jefferson Medical College, Phila.


### Art. XV.—NOTES ON THE ANATOMY OF THE INDIAN ELEPHANT.

### I.—Some Observations on the Brain.

BY PROF. HENRY C. CHAPMAN, M. D.,
*Jefferson Medical College, Phila.*

Though the brain of the elephant has been more or less described, among others by Perrault,[1] Moulins,[2] Blair,[3] Stukeley,[4] Duvernoy,[5] Camper,[6] Cooper,[7] Leuret,[8] the opportunity does not often present itself of examining an elephant's brain in a fresh condition, the following observations, based upon the study of that of the young male elephant that recently died

1. Mém pour servir à l'hist des animaux, 3e partie, p. 135. Mem de l'Acad des sciences 1666-1699 t III.
2. An anatomical account of an elephant, 1682.
3. Trans. of the Royal Society, vol. 27, vol. 30.
4. Essay towards the anatomy of an elephant, 1722.
5. Mem de l'Acad de St. Petersburg, t IV, 1729.
6. Description d'un elephant, male, 1802.
7. Referred to in Tiedeman Brain of Negro Phil Trans, vol. 1836.
8. Anatomie Comparee du Systeme Nerveux, Paris, 1839. Tome, 1, pp. 394, 446.

at the Philadelphia Zoological Garden, so far as its softened state permitted, may not appear superfluous. The brain was found to weigh, immediately after removal from the cranial cavity, without the dura mater, which was very thick, 10½ pounds, exceeding in weight any of the brains examined by the anatomists just referred to, the brain of the elephant weighing, according to Perrault, Moulins and Cooper, for example, 9 pounds, 10 pounds, 8 pounds 1 ounce, respectively. While the brain in the elephant is therefore absolutely heavier and larger than that of the grampus (*Delphinus griseus*), weighing 7 pounds, as found by the author, or of the whale (*Balæna mysticetus*), 5 pounds, according to Rudolphi,* or that of man, 49½ ounces, it is relatively to the bulk of the huge beast a small brain, even if the animal be a young one, especially when the great size of the head is taken into consideration. In removing the brain from the skull, the author was much impressed with the size of the cerebellum, which was literally enormous, being about equal in size and weight to one of the hemispheres and entirely uncovered by the latter, its surface being greatly extended through the development of the lateral lobes and very much convoluted through the presence of numerous and deep fissures. This remarkable development of the cerebellum is an interesting confirmation of the view, based upon experiment and pathology, that the cerebellum is the great reflex centre in which afferent impressions are converted into efferent ones by which equilibrium is maintained and muscular actions co-ördinated. Such being the function of the cerebellum, it is obvious why, in the elephant, in which the power of muscular co-ordination is so perfect, that this portion of the encephalon is so much developed. The *pons varolii*, corresponding in size with the development of the lateral lobes of the cerebellum, is especially interesting, as giving apparent origin to the fifth pair of nerves, which, as might be expected, are very large, being developed in proportion to the size of the trunk, or proboscis, which, through their nasal branches, they supply. As regards the medulla oblongata, the large size and prominence of the

---

* Grundriss der Physiologie Berlin, 1823, Band II, S 12.

olivary bodies are especially noticeable. With reference to the hemispheres of the elephant's brain, the most noticeable feature is not only their size, but the extent to which their surface is convoluted, the fissures exceeding in number, depth and complexity even those of the brain of man or of the cetacea, thereby supplementing possibly the deficiency in the gray matter of the cortex arising through the imperfect development of the posterior lobes of the cerebrum, the cerebellum, as already mentioned, being entirely uncovered by the latter. Owing to the softened condition of the hemispheres, due to previous inflammation, and which involved the enormous frontal sinuses as well (to which the irritability of the elephant during life was, no doubt, due), it was found impossible to remove satisfactorily the pia mater, even after the brain had been immersed in zinc chloride solution for several days, and which rendered the identification of the convolutions and fissures both difficult and doubtful. Their general disposition, however, appeared to be about the same as that observed by the author in the elephant brains preserved in the museum of the Jardin des Plantes, Paris, and kindly placed at his disposition for study by Prof. Ponchet. As regards the fissures of the hemispheres, there were two, however, about whose identification there could be no doubt—the fissures of Sylvius and Rolando. Admitting that the well marked fissure descending almost to the fissure of Sylvius, though not reaching it, is homologous with the fissure of Rolando or central fissure of the brain of man, and other primates, chimpanzee orang, &c., then the anterior and posterior convolutions, separated by the same, may be appropriately designated as the anterior and posterior central convolutions, and the four tiers of frontal convolutions passing from the anterior central convolution forward as the superior, middle, inferior, frontal and orbital convolutions.

Whether these convolutions in the elephant's brain be homologous or not with those of man and ape may be questioned, but there can be but little doubt that they have no homologues in the brains of the lower mammalia, the anterior or frontal lobe being therefore remarkably well developed in the brain of the elephant. As regards the posterior parietal portion of the hemisphere one well marked fissure presented itself and which possibly corresponds to the inter parietal fissure of the human brain. As this portion of the hemisphere was more particularly softened—any fuller identification was impossible. On the mesial surface of the hemisphere the gynes fornicatus and calloso-marginal fissures were well marked. Notwithstanding that the posterior lobes of the hemisphere in this elephant's brain are but little developed, if not absent altogether, the lateral ventricle was very large, extending far forward, and in its depth and width reminding the author of that of the brain of the manatee, with which animal the elephant through the dinotherium has other affinities as well. The thalami optici and corpora striata were well developed, particularly the latter, as also the hippocampus major; the posterior cornu of the lateral ventricle with the hippocampus minor were not, however, observed; the absence of the latter may have been due though to desintegration. From the fact, however, of the cerebellum being entirely uncovered, in all probability the posterior lobe, posterior cornu and hippocampus minor are normally absent in the elephant's brain. It was noticed that the optic lobes while present were small in comparison to the size of the remaining basal ganglia. In concluding this necessarily long notice of the elephant's brain, it will be observed that although the cerebellum is uncovered, that the brain of the elephant is nevertheless of a high order of brain as shown by the development of its frontal lobes and the number, depth and complexity of its convolutions and fissures. This is as might be expected on the supposition that the cerebral hemisphere and the frontal portions of the same more especially constitute the physical substrata of the intellectual powers. Since the intelligence of the elephant from time immemorial has been proverbial, indeed Aristotle[1] among the ancients regarded the elephant as the most intelligent of animals, and while this opinion would not be shared by all naturalists of the present day, many considering the intelligence of the ape, dog, horse and perhaps beaver, as of as high an order as that of the elephant. Never-

(1) Hist Animalia Lib IX Cap XXXIII.

theless there can be no question that the remarkable mental powers of the elephant are developed proportionately to those of its great brain.

